# Alkyne-Tagged Apigenin, a Chemical Tool to Navigate Potential Targets of Flavonoid Anti-Dengue Leads

**DOI:** 10.3390/molecules26226967

**Published:** 2021-11-18

**Authors:** Kowit Hengphasatporn, Benyapa Kaewmalai, Somruedee Jansongsaeng, Vishnu Nayak Badavath, Thanaphon Saelee, Thamonwan Chokmahasarn, Tanatorn Khotavivattana, Yasuteru Shigeta, Thanyada Rungrotmongkol, Siwaporn Boonyasuppayakorn

**Affiliations:** 1 Center for Computational Sciences, University of Tsukuba, 1-1-1 Tennodai, Tsukuba, Ibaraki 305-8577, Japan; heng.kowit@gmail.com (K.H.); shigeta@ccs.tsukuba.ac.jp (Y.S.); 2 Applied Medical Virology Research Unit, Department of Microbiology, Faculty of Medicine, Chulalongkorn University, Bangkok 10330, Thailand; benyapa.k1996@gmail.com (B.K.); vishnu.niper@gmail.com (V.N.B.); thanaphon.saelee@gmail.com (T.S.); 3 Interdisciplinary Program in Microbiology, Graduate School, Chulalongkorn University, Bangkok 10330, Thailand; 4 Center of Excellence for Natural Product, Department of Chemistry, Faculty of Science, Chulalongkorn University, Pathumwan, Bangkok 10330, Thailand; somruedee.jan@gmail.com (S.J.); 6133136023@student.chula.ac.th (T.C.); tanatorn.k@chula.ac.th (T.K.); 5 Structural and Computational Biology Research Unit, Department of Biochemistry, Faculty of Science, Chulalongkorn University, Bangkok 10330, Thailand; thanyada.r@chula.ac.th; 6 Program in Bioinformatics and Computational Biology, Graduate School, Chulalongkorn University, Bangkok 10330, Thailand

**Keywords:** alkyne-tagged flavonoid, dengue virus, drug discovery, alkyne-azide cycloaddition, flavone, target identification

## Abstract

A flavonoid is a versatile core structure with various cellular, immunological, and pharmacological effects. Recently, flavones have shown anti-dengue activities by interfering with viral translation and replication. However, the molecular target is still elusive. Here we chemically modified apigenin by adding an alkyne moiety into the B-ring hydroxyl group. The alkyne serves as a chemical tag for the alkyne-azide cycloaddition reaction for subcellular visualization. The compound located at the perinuclear region at 1 and 6 h after infection. Interestingly, the compound signal started shifting to vesicle-like structures at 6 h and accumulated at 24 and 48 h after infection. Moreover, the compound treatment in dengue-infected cells showed that the compound restricted the viral protein inside the vesicles, especially at 48 h. As a result, the dengue envelope proteins spread throughout the cells. The alkyne-tagged apigenin showed a more potent efficacy at the EC_50_ of 2.36 ± 0.22, and 10.55 ± 3.37 µM, respectively, while the cytotoxicities were similar to the original apigenin at the CC_50_ of 70.34 ± 11.79, and 82.82 ± 11.68 µM, respectively. Molecular docking confirmed the apigenin binding to the previously reported target, ribosomal protein S9, at two binding sites. The network analysis, homopharma, and molecular docking revealed that the estrogen receptor 1 and viral NS1 were potential targets at the late infection stage. The interactions could attenuate dengue productivity by interfering with viral translation and suppressing the viral proteins from trafficking to the cell surface.

## 1. Introduction

Mosquito-borne viral infections are significant burdens to tropical and subtropical countries because of global warming, increasing urbanization, and deforestation [1]. Dengue virus (DENV) is a key player responsible for 67–136 million reported cases every year [2]. Moreover, chikungunya and Zika cases have increased rapidly since 2013 [3,4]. Currently, there is no specific treatment. A vaccine for dengue was commercially available but recent reports have suggested vaccine-induced, antibody-dependent enhancement (ADE) in children less than 9 years old [5], thus limiting the vaccine-eligible population. There is no antiviral drug despite tremendous efforts in drug discovery and development [6]. Nine compounds have been in dengue clinical trials, but none of them have been FDA-approved for commercialization.

Natural products are one of the rich sources of biologically active scaffolds. Recent studies and reviews suggested flavonoid as a significant anti-flaviviral lead with mild toxicity in the cell-based system [7,8,9,10,11]. Quercetin [8], diisopropyl chrysin-7-yl phosphate [12], fisetin [13], glabranine and 7-*O*-methyl-glabranine [14] inhibited dengue RNA replication in cell-based assays. Agathisflavone, quercitrin, and isoquercitrin noncompetitively inhibited dengue virus NS2B-NS3 protease in vitro [15], whereas cyclohexenyl chalcone derivatives competitively inhibited the NS2B/NS3 protease [16]. Baicalein inhibited dengue virus during adsorption and intracellular replication [17]. Its metabolite, baicalin, inhibited dengue virus replication by targeting nonstructural proteins [7].

Despite viral targets, several cellular mechanisms have been reported as targets of flavonoid compounds. The flavonoids were also reported to generate transient DNA or protein adducts and interfere with radical scavenging activities. Therefore, specific interaction between ligand and amino acid residues should be used to identify the molecular target [18]. Investigating the subcellular distribution by addition of a chemical tag and visualization using an alkyne-azide click reaction could be a potential method to elucidate their molecular targets. In principle, a terminal alkyne was incorporated into the active leads, introduced into virus-infected cells, and visualized by fluorescence-coupled cycloaddition reaction under confocal microscopy. Subcellular localization would be highly beneficial for further molecular target identification. In this study, we focused on the proof of principle of utilizing a newly synthesized alkyne-tagged apigenin, a flavone containing the B-ring hydroxyl group, as a tool to visualize the molecular target in virus-infected cells.

## 2. Results

### 2.1. Synthesis of Alkyne-Tagged Flavone Compound

**Figure 1:**
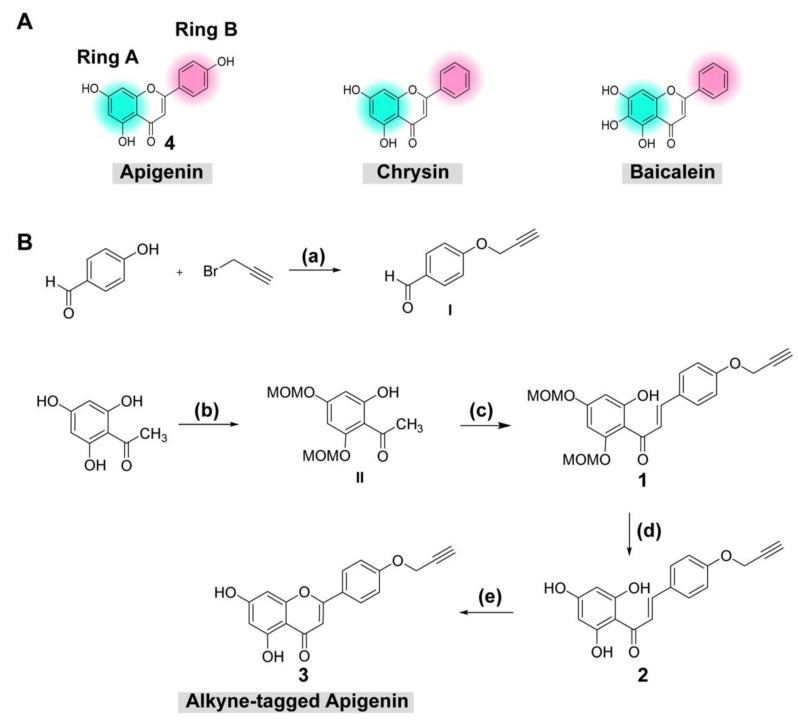
(**A**) Chemical structure of apigenin and (**B**) reagents and conditions for synthesis of alkyne-tagged apigenin: (a) K_2_CO_3_ (3.0 equiv), acetone, 90 °C, 4 h (quantitative yield); (b) MOM-Cl (3.5 equiv), K_2_CO_3_ (2.5 equiv), acetone, 90 °C, 4 h (20% yield), (c) **I** (2.0 equiv), aq 50% KOH, EtOH, rt, 24 h (57% yield); (d) 15% HCl/*i*-PrOH, rt, 30 min (38% yield); (e) I_2_ (cat.), DMSO, 90 °C, 2 h (20% yield).

Previous reports confirmed that flavones (e.g., chrysin derivatives and baicalein) were potential antivirals [9,15], but the molecular targets were still elusive. Evidence suggested that the target should be involved in viral translation/replication as the compounds inhibited the viral replicon or a self-replicating RNA element inside the cell. However, chrysin and baicalein lack the B-ring interacting moiety for an alkyne modification. Therefore, a structurally similar compound with a B-ring hydroxyl group, apigenin, was chosen for the chemical modification (Figure 1A). The terminal alkyne was introduced into the B-ring hydroxyl group (Figure 1A). In this work, the alkyne-tagged apigenin was synthesized according to the synthetic sequence outlined (Figure 1B). First, the alkyne group was introduced by the reaction between 4-hydroxy benzaldehyde with propargyl bromide to give **I** in good yield (step a) [19]. Next, the protection of the hydroxyl groups of the commercially available 2,4,6-trihydroxyacetophenone with methoxymethyl chloride (MOM-Cl) yielded MOM-protected intermediate II (step b) [20], which was further combined with I under basic conditions through aldol condensation leading to the formation of compound 1 (step c) [21]. The MOM-deprotection of 1 under acidic conditions gave 2 (step d) [20], which was then subjected to the conditions for chalcone cyclization using a catalytic amount of iodine in DMSO (step e) [22], yielding the desired alkyne-tagged apigenin 3. All compounds were identified using ^1^H and ^13^C-NMR, and HRMS for the final product 3 (Supplementary Figures S2–S7).

### 2.2. Cell-Based Antiviral Assays

Efficacies and cytotoxicities were tested with both original and alkyne-tagged compounds (Table 1). Results showed that alkyne-tagged apigenin (**3**) inhibited DENV2 infectivity at least tenfold compared to the original apigenin (**4**), with EC_50_s of 2.36 ± 0.22 µM and 10.55 ± 3.37 µM, respectively. On the contrary, the cytotoxicities were similar at 70.34 ± 11.79 µM and 82.82 ± 11.68 µM, respectively. Our results with the original apigenin were similar to those previously reported [23]. The original apigenin at 40 µM was previously reported to suppress >50% of DENV3 titer, and around 30% of DENV2 (Table 1). Therefore, it was possible that the alkyne-tagged apigenin exhibited additional interactions or increased permeability from the originals.

**Figure 2:**
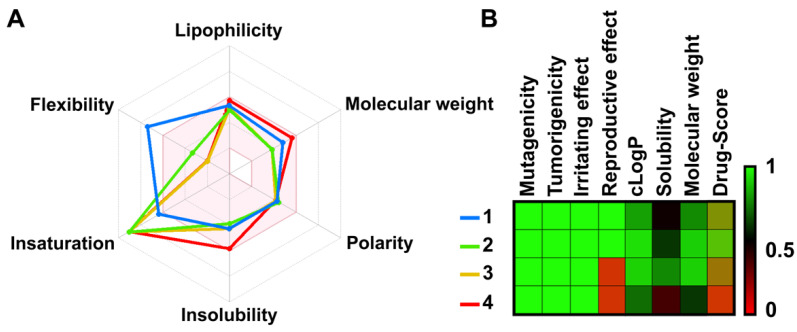
(**A**) Bioavailability radar was computed by 2D chemical structures according to their physicochemical properties such as flexibility, lipophilicity, molecular weight, polarity, water solubility, and saturation. An accepted region is represented as a pink area. (**B**) The toxicity risks and pharmacological properties of designed compounds were predicted by OSIRIS Property Explorer. Each value ranges from 0 to 1 (red, black, green).

The physicochemical analysis using the SwissADME program found both apigenins (**3** and **4**) and chalcones (**1** and **2**) were lipophilic and soluble within the acceptable range [24]. The alkyne-tagged and original apigenin (**3** and **4**) showed 0/1 (triple bond) and 0/0 PAINS/BRENK scores, respectively (Supplementary Figure S8) [24]. Therefore, the compounds were predicted to be freely permeable across cellular and subcellular membranes (Figure 2A) [24]. The overall drug score was acceptable for both apigenins (Figure 2B) [25].

**Figure 3:**
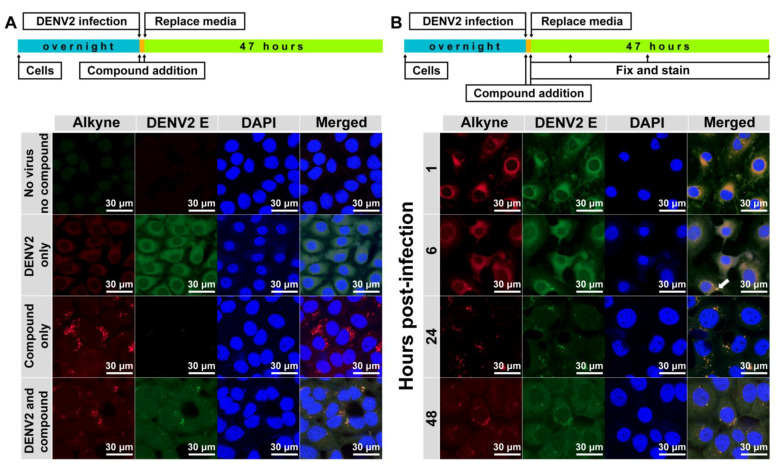
Subcellular localization (**A**,**B**) of alkyne-tagged apigenin. DENV2 NGC (MOI of 1) and the newly synthesized alkyne-tagged apigenin (10 µM) were added to LLC/MK2 (10^5^) cells in a 12 mm presterilized coverslip and incubated for 48 h, unless indicated otherwise, at 37 °C under 5% CO_2_. Cells were fixed and permeabilized by acetone before washing with 1% bovine serum albumin (BSA) in phosphate buffer saline (PBS) and staining with Click-iT^®^ reaction cocktail (Thermo Fisher, Waltham, MA, USA) D1-4G2-4-15 antibody, followed by goat anti-mouse conjugated with FIT-C (Santa Cruz Biotechnology, Santa Cruz, CA, USA) and DAPI. The slide was air-dried and visualized by confocal microscope model LSM800 with Airyscan (Zeiss, Oberkochen, Germany).

The 10 µM alkyne-tagged apigenin (**3**) was added into the virus-infected cells (MOI of 1) and incubated for 48 h before visualization using a confocal microscope model LSM800 with Airyscan (Zeiss, Oberkochen, Germany) (Figure 3A). Results were compared with the DENV2 infection without a compound (DENV2 only) and the compound treatment without the infection (compound **3** only). The compound signal was concentrated in vesicles regardless of the viral infection. However, the distribution of DENV2 E protein was different in the presence or absence of the compound. Furthermore, the viral protein signal was detected mainly throughout the cytoplasm in DENV2 only, whereas the DENV2 E was confined in the vesicles in the presence of the compound. This result suggested a possible interaction between the compound and viral proteins that could change the viral protein distribution and inhibit virion production. However, this finding was a snapshot of a late infection (48 h), which might not represent the overall compound-virus interactions that could differ in other stages of the viral life cycle, especially at early time-points. We then terminated the drug-addition experiment in dengue-infected cells at 1, 6, 24 and 48 h after infection (Figure 3B). Results showed that the compound and dengue protein mainly colocalized at the perinuclear cytoplasm early after infection at 1–6 h post-infection before moving to the vesicles at 24–48 h post-infection. Interestingly, a vesicle signal was noticed (Figure 3B, arrow) at 6 h post-infection suggesting a transition of localization could start at this time point.

### 2.3. Potential Targets of Apigenins

**Figure 4:**
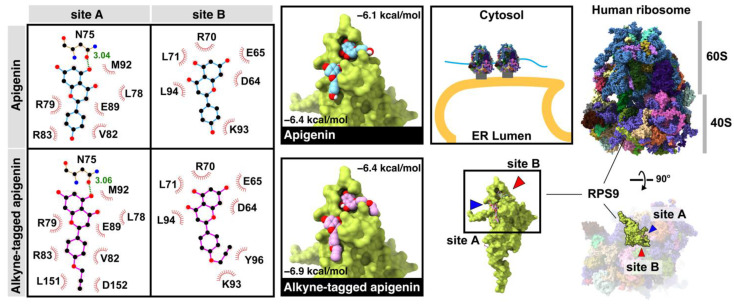
The binding interaction and conformation of apigenin and alkyne-tagged apigenin on the ribosomal protein S9 (RPS9) at sites A and B.

A ribosomal protein S9 (RPS9) protein was reported as a target using an apigenin-fixed bead and MALDI-TOF MS methods and orthogonally proven by a RPS9-targeting siRNA knockdown experiment [26]. The RPS9 was retrieved from the protein database (PDB code: 6OM7) for molecular docking. Results showed that apigenin and alkyne-tagged apigenin bound to two sites (A and B) with similar binding residues (site A: N75, L78, R79, V82, R83, E89, M92; site B: D64, E65, R70, L71, K93, L94) (Figure 4). Moreover, the alkyne-tagged apigenin showed additional binding residues (site A: L151, D152; site B: Y96), which could explain the slightly lower binding energy and lower EC_50_ than the original apigenin. Moreover, the ribosomal proteins were upregulated exclusively for 6 h after infection [27], corresponding to our compound localization in the early time points.

**Figure 5:**
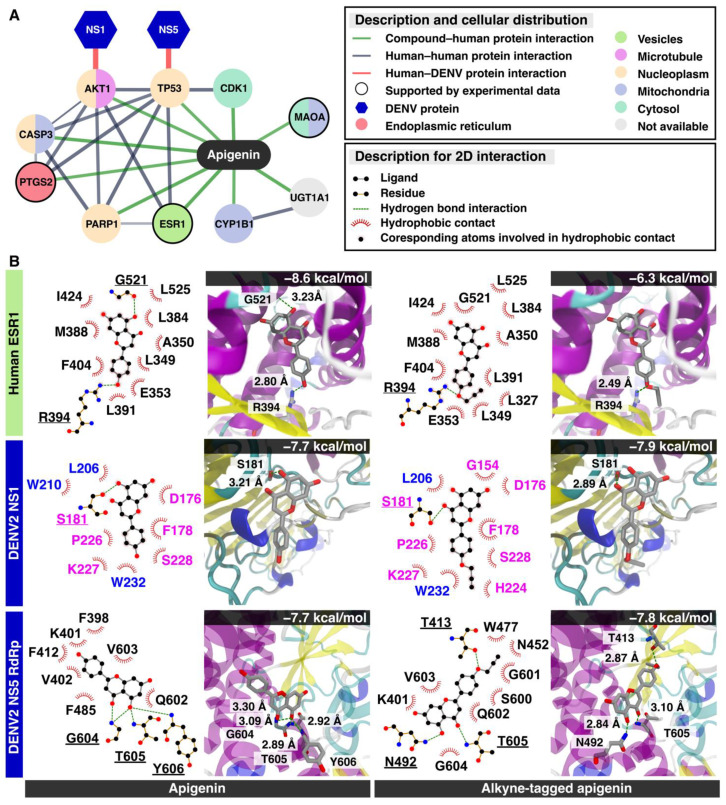
Possible viral targets for apigenin and alkyne-tagged apigenin. (**A**) The integration between the network-based method from the STITCH database and the Human Protein Atlas database. The line thickness indicates the strength of data support (the details are shown in Table S1 in Supporting Materials), and only high confidence predicted proteins are displayed on this network. The edges between predicted protein—human human—protein interactions, DENV–human proteins interactions, and chemical–protein interactions—are represented by grey, red, and green colors, respectively. The color of each circle node shows the cellular localization of each human protein, and the only node verified by experimental data is revealed as the black circle border. DENV protein shows as the hexagon node. (**B**) The molecular docking results in 2D interaction and 3D visualization between apigenin, alkyne-tagged apigenin, and possible targets: the human ESR1 protein, DENV NS1, and NS5 RdRp.

In late time points, the network-based method from the STITCH database [28] revealed that the apigenin could directly relate to 10 cellular proteins, estrogen receptor 1 (ESR1), UDP glucuronosyltransferase 1 family (UGT1A1), monoamine oxidase A (MAOA), tumor protein p53 (TP53), cyclin-dependent kinase 1 (CDK1), caspase 3 (CASP3), prostaglandin-endoperoxide synthase 2 (PTGS2), v-AKT murine thymoma viral oncogene homolog 1 (AKT1), poly (ADP-ribose) polymerase 1 (PARP1), and cytochrome P450 (CYP1B1) (Figure 5A and Table S1 in Supplementary Materials). The results showed that only three human targets, MAOA, PTGS2, and ESR1, were confirmed by the biological experiments (black circle node in Figure 5). These proteins were located for their subcellular distribution using the Human Protein Atlas database [29] labeled by different colors in each node. Only ESR1 was located mainly in the cellular vesicles. ESR1 inhibition attenuated the flaviviral replication [30] from the molecular mechanism aside from the classical estrogen receptor signaling pathway. In addition, the ten cellular proteins previously characterized (Figure 5A) were tested to find whether they were related to DENV replication using homopharma and network-based analysis [31]. The network analysis suggested that AKT1 and TP53 could be associated with viral nonstructural proteins (NS1 and NS5), respectively. The NS1 viral protein plays an essential role in host–pathogen interaction as it is the most abundant viral protein circulating in the bloodstream. In the confocal microscopy, the dimeric NS1 resided in the ER lumen [32,33], whereas the DENV NS5 protein located mainly in ER-derived spherules and in the nucleus [34,35]. The DENV NS5 protein consists of an N-terminal methyltransferase (MTase), a C-terminal RNA-dependent RNA polymerase (RdRp), and also suppresses the STAT-2 interferon signaling [36].

The molecular docking of apigenin with three predicted proteins including ESR1, NS1, and NS5 RdRp was performed (Figure 5B). The MTase was excluded from the subsequent molecular docking study because apigenin might be not able to inhibit the NS5 MTase by a high-throughput enzyme assay [37]. The docking results suggested that the three proteins could bind to apigenin and alkyne-tagged apigenin at the same binding region. First, both apigenins attached to ESR1 in the same position and the alkyne moiety were exposed to the subsequent cycloaddition reaction. The finding suggested that the alkyne tag did not interfere with the compound binding. However, the alkyne-tagged apigenin only showed a single hydrogen bond at the residue R394 (2.80 Å) (−6.3 kcal/mol), whereas the apigenin had two residues, G521 (3.23 Å) and R394 (2.80 Å) (−8.6 kcal/mol) to the native binding site of the ESR1 protein. Therefore, the alkyne-tagged apigenin could bind to ESR1 but in a weaker way than the original apigenin and the substrate (estradiol, −10.6 kcal/mol) (Figure S1A in Supplementary Materials) [38]. However, the similar binding poses of both apigenins suggested that the alkyne-tagged compound could represent the original apigenin [39]. Next, the dimeric NS1 bound to both apigenins in an identical position and energy (−7.7 kcal/mol) at the groove between loop (β1 and β2) and (β10 and β11) of the β-roll and β-ladder, respectively. The alkyne moiety was also exposed to the surface, suggesting a successful cycloaddition reaction. Moreover, NS1 localized at ER lumen and transport vesicles, implying a colocalization potential with alkyne-tagged apigenin during late infection. Last, the NS5 interacting residues were in the RNA binding groove with a similar energy score (−7.7 to −7.8 kcal/mol). However, the binding poses of the alkyne-tagged apigenin differed from the original, and the alkyne moiety was found submerged into the RNA-binding groove, preventing a subsequent cycloaddition from occurring. Therefore, the NS5 was unlikely to be the compound target based on the molecular docking study. In summary, the alkyne-tagged apigenin could represent the ESR1 and NS1 as potential targets of the apigenin during late infection based on the molecular docking study.

Collectively, this is a proof of concept of an alkyne-tagged flavonoid for subcellular localization tracking and tracing inside the virus-infected cells. This presence of the compound changed the subcellular localization of the viral protein during the late time points of the infection. Moreover, the molecular targets could be predicted using the network-based method and molecular docking. The apigenin should attenuate dengue productivity by interfering with viral translation and suppressing the viral proteins from trafficking to the cell surface. Further applications of this chemical tag would be coupling the cycloaddition reaction with the streptavidin-biotin pulldown and identify the protein target by Western blot or LC-MS/MS.

## 3. Discussion

Undoubtedly, flavonoids have proven effective against many RNA viruses [40]. Each flavonoid interferes with various stages of viral replication through multiple mechanisms (e.g., viral inactivation, viral fusion, viral translation/replication, autophagy, anti-inflammation, etc.). Moreover, flavonoids could establish a broad range of safety profiles since most are mildly toxic in vivo. This study focused on implementing an alkyne-azide cycloaddition technology to a flavone and used it as a tool to visualize the compound localization inside the cells. Apigenin was chosen as a flavone derivative because its existing hydroxyl group on the B-ring was suitable for a terminal alkyne modification. Although apigenin itself was a moderately potent inhibitor at the EC_50_ of 10.55 ± 3.37 µM, it represented its chemical relatives like chrysins and baicaleins (Figure 1A), which were strong DENV inhibitors [9,12,17] but lacked the B-ring hydroxyl group for alkyne addition. The newly synthesized alkyne-tagged apigenin identity was verified by ^1^H-NMR, and ^13^C-NMR (Figures S2–S7 in Supplementary Materials). The compound at 10 µM was introduced into the virus-infected cells based on the efficacy and cytotoxicity profile. According to previous reports, viral translation is a primary target during early infection [9,12,17]. The compound is located at the perinuclear region and colocalized with a dengue protein for 1–6 h after infection (Figure 3B). Obviously, dengue replication requires a ribosomal machinery for its translation and the ribosomal proteins were strongly upregulated during early infection. We confirmed the ribosomal protein RPS9 as one of the targets of alkyne-tagged apigenin (Figure 4) previously characterized by a pulldown experiment [26]. The binding energy of both original and alkyne-tagged apigenin with the RPS9 was similar at −6.9 to −6.4 and −6.4 to −6.1 kcal/mol, respectively. Therefore, RPS9 should be one of the molecular targets of the apigenin and contributes to the inhibition of the viral translation in the early phase.

The alkyne apigenin shifted its compartment during late time points to vesicular structures regardless of the viral infection (Figure 3A). Moreover, the DENV signal also shifted its localization into the vesicle-like structure in the presence of the compound. Interestingly, the DENV signal still dispersed throughout the cytosol at 48 h after infection in the absence of the alkyne apigenin (Figure 3A). The estrogen receptor (ESR) 1 was suspected as it was the only protein interacting with the apigenin and located in vesicles (Figure 4). Moreover, a previous report suggested that targeting the ESR evidently inhibited the flaviviral replication [30]. The molecular docking revealed that the alkyne-tagged apigenin could bind to ESR1 with a weaker interaction than the original apigenin. Therefore, the ESR1 could be a potential target using this chemical tag method. Two viral proteins, NS1 and NS5 RdRp, were previously reported as potential binding partners of AKT and TP53 targets of apigenin, respectively. The NS1 locates in the ER lumen and transport vesicles, whereas the NS5 is located in the ER-derived spherules and nucleus. The binding energy to both apigenins to NS1 and NS5 were similar at −7.7 to −7.9 kcal/mol. Still, the compound alignment excluded NS5 from the potential target as the alkyne moiety was not accessible for cycloaddition. In summary, the cellular ESR1 and viral NS1 were suggested as possible targets during late infection.

## 4. Materials and Methods

### 4.1. Materials and Instrumentation for Chemical Synthesis

All reagents and solvents were obtained from Sigma-Aldrich (St. Louis, MO, USA), TCI chemicals (Tokyo, Japan), and Merck (Darmstadt, Germany). All solvents for column chromatography (RCI Labscan, Samutsakorn, Thailand) were distilled before use. Reactions were monitored by thin-layer chromatography (TLC) using aluminum Merck TLC plates coated with silica gel 60 F254. Normal phase column chromatography was performed using silica gel 60 (0.063–0.200 mm, 70–230 mesh ASTM, Merck, Darmstadt, Germany). Proton (^1^H) and carbon (^13^C) nuclear magnetic resonance spectra were recorded on a Bruker Advance, (III) 400 WB Fällanden, Switzerland and JEOL JNM-ECZ500/S1 (500 MHz) spectrometers. Chemical shifts were expressed in parts per million (ppm), *J* values were in Hertz (Hz).

### 4.2. Synthetic Procedure of Alkyne Tag Compounds

*4-(Prop-2-yn-1-yloxy) benzaldehyde* (I): the title compound was synthesized using a modified procedure from the literature [16]. To a solution of 4-hydroxybenzaldehyde (300 mg, 2.45 mmol, 1.0 equiv) and potassium carbonate (1 g, 7.35 mmol, 3 equiv) in acetone (8 mL), propargyl bromide (557 µL, 7.35 mmol, 3 equiv) was added dropwise, and then the suspension was refluxed for 4 h at 90 °C. After cooling, the solid was filtered off, and the filtrate was concentrated to afford the title compound as a beige solid (590 mg, 3.68 mmol, quantitative yield). The crude product was used without further purification. ^1^H-NMR (500 MHz, DMSO-*d6*): δ 8.98 (s, 1H, C*H*O), 6.99 (d, 2H, *J* = 8.8 Hz, Ar*H*), 6.27 (d, 2H, *J* = 8.6 Hz, Ar*H*), 4.04 (d, 2H, *J* = 2.3 Hz, -OC*H*_2_C≡CH), 1.60 (t, 1H, *J* = 2.6 Hz, -C≡C*H*); ^13^C-NMR (126 MHz, DMSO-*d6*): δ 191.47, 162.06, 131.75, 130.18, 115.35, 78.89, 78.56, 55.90.

*1-(2-Hydroxy-4,6-bis(methoxymethoxy)phenyl)thenone* (II): the title compound was synthesized using a modified procedure from the literature [19]. To a solution of 2,4,6-trihydroxyacetophenone monohydrate (300 mg, 1.78 mmol, 1.0 equiv) and K_2_CO_3_ (615 mg, 4.45 mmol, 2.5 equiv) in acetone (8 mL), MOM-Cl, (473 µL, 6.23 mmol, 3.5 equiv) was added dropwise, and then the suspension was refluxed overnight at 50 °C. After cooling, the K_2_CO_3_ was filtered off, and the crude product was concentrated and purified by column chromatography on silica gel (eluent: 2% EtOAc:hexanes to 5% EtOAc:hexanes) to afford the title compound as a white solid (91 mg, 0.355 mmol, 20% yield). ^1^H-NMR (500 MHz, CDCl_3_): δ 6.25 (d, 1H, *J* = 2.3 Hz, Ar*H*), 6.23 (d, 1H, *J* = 2.3 Hz, Ar*H*), 5.24 (s, 2H, -OC*H*_2_O-), 5.15 (s, 2H, -OC*H*_2_O-), 3.50 (s, 3H, -OC*H*_3_), 3.48 (s, 3H, -O*H*_3_), 2.05 (s, 3H, C*H*_3_CO); ^13^C-NMR (126 MHz, CDCl_3_): δ 203.31, 166.90, 163.54, 160.44, 107.00, 97.22, 94.54, 94.08, 56.77, 56.51, 33.08. ^1^H and ^13^C data are consistent with the literature values [41].

*(E)-1-(2-Hydroxy-4,6-bis(methoxymethoxy)phenyl)-3-(4-(prop-2-yn-1-yloxy)phenyl)prop-2- en-1-one* (**1**): the title compound was synthesized using a modified procedure [18]. A solution of 4-(prop-2-yn-1-yloxy) benzaldehyde (114 mg, 0.71 mmol, 2.0 equiv) in EtOH (1.5 mL) was added into a solution of 1-(2-hydroxy-4,6-bis(methoxymethoxy)phenyl)thenone hydroxybenzaldehyde (91 mg, 0.355 mmol, 1.0 equiv) in EtOH (1.5 mL). Then, 50% KOH solution was slowly added into the mixture at 0 °C. The reaction mixture was stirred overnight at room temperature. After the reaction was complete, the solution was quenched with 1M HCl. The mixture was extracted with EtOAc, washed with water and brine. The combined organic layers were dried over MgSO_4_, filtered, concentrated, and purified by column chromatography on silica gel (eluent: 4:0.5:0.5 hexane:EtOAc:DCM) to afford the title compound as a yellow solid (80 mg, 0.20 mmol, 57% yield). ^1^H-NMR (500 MHz, CDCl_3_): δ 7.83 (d, 1H, *J* = 15.5 Hz, *trans*-C*H*=CHCO-), 7.77 (d, 1H, *J* = 15.6 Hz, *trans* -CH=C*H*CO-), 7.57 (d, 2H, *J* = 8.9 Hz, Ar*H*), 7.01 (d, 2H, *J* = 8.8 Hz, Ar*H*), 6.31 (d, 1H, *J* = 2.3 Hz, Ar*H*), 6.24 (d, 1H, *J* = 2.4 Hz, Ar*H*), 5.29 (s, 2H, -OC*H*_2_O-), 5.18 (s, 2H, -OC*H*_2_O-), 4.73 (d, 2H, *J* = 2.4 Hz, -C*H*_2_C≡CH), 3.53 (s, 3H, OC*H*_3_), 3.48 (s, 3H, OC*H*_3_), 2.56 (t, 1H, *J* = 2.4 Hz, -CH_2_C≡C*H*); ^13^C-NMR (126 MHz, CDCl_3_): δ 192.91, 167.39, 163.43, 159.92, 159.32, 142.43, 130.08, 129.10, 125.54, 115.39, 107.63, 97.60, 95.24, 94.85, 94.14, 78.14, 76.07, 56.96, 56.55, 55.91.

*(E)-3-(4-(Prop-2-yn-1-yloxy)phenyl)-1-(2,4,6-trihydroxyphenyl)prop-2-en-1-one* (**2**): the title compound was synthesized using a modified procedure [17]. A solution of **1** (220 mg, 0.55 mmol, 1.0 equiv) was dissolved in 15% HCl/*i*PrOH (1:5, 33 mL). Then, the solution was stirred at room temperature for 4 h and monitored by TLC. After the reaction was complete, the reaction mixture was quenched with saturated NaHCO_3_ and extracted with EtOAc. The combined organic layers were dried over anhydrous Na_2_SO_4_, concentrated, and purified by column chromatography on silica gel (eluent: 5% to 20% EtOAc:hexane) to afford the title compound as a yellow solid (65 mg, 0.21 mmol, 38% yield). ^1^H-NMR (500 MHz, acetone-*d6*): δ 8.16 (d, 1H, *J* = 15.6 Hz, *trans*-C*H*=CHCO-), 7.76 (d, 1H, *J* = 15.6 Hz, *trans*-CH=C*H*CO-), 7.67 (d, 2H, *J* = 8.9 Hz, Ar*H*), 7.08 (d, 2H, *J* = 8.8 Hz, Ar*H*), 5.96 (s, 2H, Ar*H*), 4.86 (d, 2H, *J* = 2.4 Hz, -C*H*_2_C≡CH), 3.13 (t, 1H, *J* = 2.0 Hz, -CH_2_C≡C*H*); ^13^C-NMR (126 MHz, acetone-*d6*): δ 192.34, 164.95, 164.86, 159.48, 141.67, 129.99, 129.07, 125.70, 115.38, 104.83, 95.21, 78.54, 76.55, 55.59.

*5,7-Dihydroxy-2-(4-(prop-2-yn-1-yloxy)phenyl)-4H-chromen-4-one* (**3**): the title compound was synthesized using a modified procedure [21]. A solution of iodine (0.8 mg, 0.003 mmol, 0.05 equiv) in dry DMSO (454 µL) was added into compound 2 (20 mg, 0.064 mmol, 1.0 equiv) in a round bottom flask. Then, the reaction mixture was stirred at 90 °C for 6 h and monitored by TLC. The reaction mixture was quenched with ice water, extracted with EtOAc, and washed with 10% Na_2_S_2_O_3_. The organic layers were dried over anhydrous Na_2_SO_4_, concentrated, and purified by preparative TLC (isocratic elution: 20% EtOAc:hexane) to give the desired title compound as a pale yellow solid (3.9 mg, 0.13 mmol, 20% yield). ^1^H-NMR (500 MHz, acetone-*d6*): δ 8.04 (d, 2H, *J* = 8.8 Hz, Ar*H*), 7.20 (d, 2H, *J* = 8.8 Hz, Ar*H*), 6.68 (s, 1H, Ar*H*), 6.55 (d, 1H, *J* = 1.9 Hz, Ar*H*), 6.26 (d, 1H, *J* = 1.9 Hz, Ar*H*), 4.93 (d, 2H, *J* = 2.3 Hz, -C*H*_2_C≡CH), 3.16 (t, 1H, *J* = 2.2 Hz, -CH_2_C≡C*H*); ^13^C-NMR (126 MHz, acetone-*d6*): δ 182.16, 167.89, 163.59, 160.66, 158.05, 136.48, 128.16, 124.40, 119.06, 115.44, 103.96, 99.19, 94.12, 78.32, 76.78, 55.74; HRMS (ESI^+^): *m*/*z* calculated for C_18_H_12_NaO_5_ [M + Na] 331.0582, found 331.0571.

### 4.3. Cell-Based Study

#### 4.3.1. Cells and Virus

LLC/MK2 (ATCC^®^ CCL-7) and C6/36 (ATCC^®^ CRL-1660) cell lines were propagated and maintained as previously described [9,42]. The DENV2 New Guinea C strain (NGC) was propagated in C6/36 cells as described in [9,42]. Cells and viruses were courtesy of Prof. Padet Siriyasatien.

#### 4.3.2. Cytotoxic Concentration (CC_50_) Test

LLC/MK2 was seeded at 10^4^ cells per well of a 96-well plate and incubated overnight. Compounds were prepared at 6–10 different concentrations in filter-sterilized dimethylsulfoxide (Merck^®^, Darmstadt, Germany) before addition to the cells. Plates were incubated for 48 h before the MTS reagent (Promega^®^, Madison, WI, USA) was added to the cells according to the manufacturer’s protocol and incubated for 4 h before analysis by spectrophotometry at *A*_450nm_. Each compound was tested in triplicate. Cytotoxic concentrations (CC_50_) were calculated using a nonlinear regression analysis and the results were reported as means and standard deviations of three independent experiments.

#### 4.3.3. Effective Concentration (EC50) Test

LLC/MK2 (5 × 10^4^) cells were seeded into each well of a 24-well plate and incubated overnight at 37 °C under 5% CO_2_. DENV2 NGC at the multiplicity of infection (MOI) of 0.1 was added to the cells for 1 h according to a previous description [9,42]. The compounds 1–4 were prepared in dimethylsulfoxide at 8–10 different concentrations and were added during and after infection. The infected cells were incubated for 72 h at 37 °C under 5% CO_2_. Supernatants were collected for analysis by plaque titration [43]. Data were plotted, and the EC_50_ values were calculated by nonlinear regression analysis. The results were reported as means and standard deviations of three independent experiments. The selectivity index was calculated from the ratio of CC_50_ and EC_50_.

#### 4.3.4. Visualization by Confocal Microscopy

LLC/MK2 (1 × 10^5^) cells were seeded into a 12 mm presterilized cover slip in each well of 24-well plates and incubated overnight. Cells were then infected with the DENV2 NGC at the MOI of 1 and the newly synthesized alkyne-tagged apigenin (10 µM) and incubated for 72 h at 37 °C under 5% CO_2_. Cells were fixed and permeabilized by acetone, 10 min before washing with 1% bovine serum albumin (BSA) in phosphate buffer saline (PBS). The Click-iT^®^ reaction cocktail was prepared within 15 min before use according to manufacturer’s protocol (Thermo Fisher, Waltham, MA, USA) and added into each sample. Cells were incubated for 30 min at room temperature protected from light. Next, a dengue protein was detected using D1-4G2-4-15 Hybridoma cell (ATCC^®^ HB-112™, ATCC, Manassas, VA, USA) for 60 min, at 37 °C protected from light, and mouse IgGκ light chain binding protein (m-IgGκ BP-FITC: sc-516140) (Santa Cruz Biotechnology, Santa Cruz, CA, USA) for 60 min, at room temperature protected from light. The nuclei were stained by DAPI for 5 min at room temperature protected from light. The slide was air-dried and visualized by confocal microscope model LSM800 with Airyscan (Zeiss, Oberkochen, Germany)

### 4.4. Molecular Target Identification

Apigenin’s target prediction was evaluated using the combination of homopharma network-based analysis, as in our previous work [31], and the molecular docking method. The chemical scaffold of apigenin was used to search for the possible target using the chemical–protein interaction network from the STITCH database [28]. The cellular localizations of the predicted protein targets were also sought using the Human Protein Atlas databases [24] and were compared to the confocal microscopy in this study. Moreover, we investigated the relationship between human and viral proteins to search for the possible viral target of apigenin using a DenvIntS PPI network-based analysis [31].

The crystal structures of ribosomal protein S9 (RPS9) (6OM7.pdb [44]), estrogen receptor 1 (1QKU.pdb [45]), DENV2 NS1 (4O6B.pdb [46]), and DENV2 NS5 RdRp (5ZQK.pdb [47]) were analyzed. The steps of system preparation and molecular docking were conducted according to a previous study [48,49]. In brief, the structure of ESR1 and NS1 was added to the missing residues using SWISS-MODEL web server tools. RPS9 was extracted from the human ribosomal protein to be a template for molecular docking. The protonation state of all ionizable residues was assigned at pH 7 using the PDB2PQR server [50]. The protein in PDB format was converted to PDBQT format by including atom types and partial atomic charges into the PDB file using ADT software [51]. Structures of apigenin and alkyne-tagged apigenin were created and fully optimized by the DFT/6-311(g) level of theory using the Gaussian16 program [52]. To identify the preferable binding regions, structural proteins were blind docked with 50 independent runs (20 hits for each run) [53]. The grid map assigned was sufficient to cover the total volume of the protein structure. Subsequently, the ligand was docked into the most favorable binding site of each protein using Autodock Vina version 1.1.2 [54]. The apigenin and alkyne-tagged apigenin with the highest binding affinities were chosen to explain the ligand–protein interactions using LigPlot+ version 2.2 software [55] and Chimera version 1.15 for visualization in the 3D diagram.

## 5. Conclusions

This is a proof-of-concept study of compound tracking and tracing inside virus-infected cells. The compound was concentrated in the perinuclear region, and the RPS9 was a potential target during early infection. The compound was concentrated in vesicle-like structures during late infection so that the target could be the ESR1 and NS1 proteins.

## 6. Patents

The alkyne-tagged apigenin synthetic scheme and the application have been filed for patenting in Thailand.

## Figures and Tables

**Table 1:** DENV2 inhibitory activity in cell-based assay of newly synthesized alkyne-tagged compounds.

Compound	EC_50_ (µM)	CC_50_ (µM)	S.I. (CC_50_/EC_50_)
**1**	3.54 ± 0.90	45.87 ± 3.05	12.98
**2**	7.81 ± 1.73	69.96 ± 14.78	8.96
**3**	2.36 ± 0.22	70.34 ± 11.79	29.80
**4**	10.55 ± 3.37	82.82 ± 11.68	7.85

Errors were calculated from three independent experiments.

## Data Availability

Not applicable.

## Supplementary Materials

Table S1: list of top 10 proteins related to apigenin and their subcellular distribution, Figure S1: A, structure overlay of estradiol and apigenins; B,C, the 3D structure of dimeric NS1 of DENV2, Figures S2–S7: ^1^H and ^13^C-NMR spectra.

## Sample Availability

The alkyne apigenin is available from the authors upon request.
