# Supplementary material for: Alkyne-Tagged Apigenin, a Chemical Tool to Navigate Potential Targets of Flavonoid Anti-Dengue Leads
Source: Molecules. 2021 Nov 18;26(22):6967. doi: 10.3390/molecules26226967 (PMC8618255; doi:10.3390/molecules26226967)
Supplement: Supplementary file 1 [file molecules-26-06967-s001.zip › molecules-1431005-supplementary.pdf]

molecules

*Supplementary Material*

# Alkyne-Tagged Apigenin, a Chemical Tool to Navigate Potential Targets of Flavonoid Anti-Dengue Leads

**Kowit Hengphasatporn [1,†], Benyapa Kaewmalai [2,3,†], Somruedee Jansongsaeng [4,†], Vishnu Nayak Badavath [2,†], Thanaphon Saelee [2], Thamonwan Chokmahasarn [4], Tanatorn Khotavivattana [4], Yasuteru Shigeta [1], Thanyada Rungrotmongkol [5,6] and Siwaporn Boonyasuppayakorn [2,*]**

[1] Center for Computational Sciences, University of Tsukuba, 1-1-1 Tennodai, Tsukuba, Ibaraki 305-8577, Japan; heng.kowit@gmail.com (K.H.); shigeta@ccs.tsukuba.ac.jp (Y.S.)
[2] Applied Medical Virology Research Unit, Department of Microbiology, Faculty of Medicine, Chulalongkorn University, Bangkok 10330, Thailand; benyapa.k1996@gmail.com (B.K.); vishnu.niper@gmail.com (V.N.B.); thanaphon.saelee@gmail.com (T.S.)
[3] Interdisciplinary Program in Microbiology, Graduate School, Chulalongkorn University, Bangkok 10330, Thailand
[4] Center of Excellence for Natural Product, Department of Chemistry, Faculty of Science, Chulalongkorn University, Pathumwan, Bangkok, 10330, Thailand; somruedee.jan@gmail.com (S.J.); 6133136023@student.chula.ac.th (T.C.); tanatorn.k@chula.ac.th (T.K.)
[5] Structural and Computational Biology Research Unit, Department of Biochemistry, Faculty of Science, Chulalongkorn University, Bangkok 10330, Thailand; thanyada.r@chula.ac.th
[6] Program in Bioinformatics and Computational Biology, Graduate School, Chulalongkorn University, Bangkok 10330, Thailand
* Correspondence: siwaporn.b@chula.ac.th
† These authors contributed equally to this work.

Overview:

Table S1. List of top10 proteins related to apigenin and their subcellular distribution

Figure S1. A structure overlay of Estradiol and apigenins B-C The 3D structure of dimeric NS1 of DENV2 with the top binding poses from 50 docking runs.

Figures S2–S7 $^{1}$H and $^{13}$C-NMR spectra

Figure S8 Pharmacokinetics, drug-likeness and medicinal chemistry by SwissADME

**Table S1.** List of top10 proteins related to apigenin and their subcellular distribution.

| Targeted Proteins | | Cellular Distribution | Prediction of Specific Actions | Experimental /Biochemical Data | Evidence Suggesting a Function Link (Combined Score) |
|---|---|---|---|---|---|
| ESR1 | estrogen receptor 1 | vesicles | activation, binding, expression | Yes | 0.961 |
| UGT1A1 | UDP glucuronosyltransferase 1 family | cytosol | inhibition | None | 0.949 |
| MAOA | monoamine oxidase A | nucleoplasm, mitochondria | activation | None | 0.947 |
| TP53 | tumor protein p53 | nucleoplasm | Not available | None | 0.944 |
| CDK1 | cyclin-dependent kinase 1 | Not available | expression | None | 0.938 |
| CASP3 | caspase 3, apoptosis-related cysteine peptidase | endoplasmic reticulum | binding | Yes | 0.877 |
| PTGS2 | prostaglandin-endoperoxide synthase 2 | nucleoplasm and microtubule | expression | None | 0.876 |
| AKT1 | v-Akt murine thymoma viral oncogene homolog 1 | mitochondria | binding, inhibition | Yes | 0.876 |
| PARP1 | poly(ADP-ribose) polymerase 1 | nucleoplasm | expression | None | 0.868 |
| CYP1B1 | cytochrome P450 | mitochondria, cytosol | binding | Yes | 0.848 |

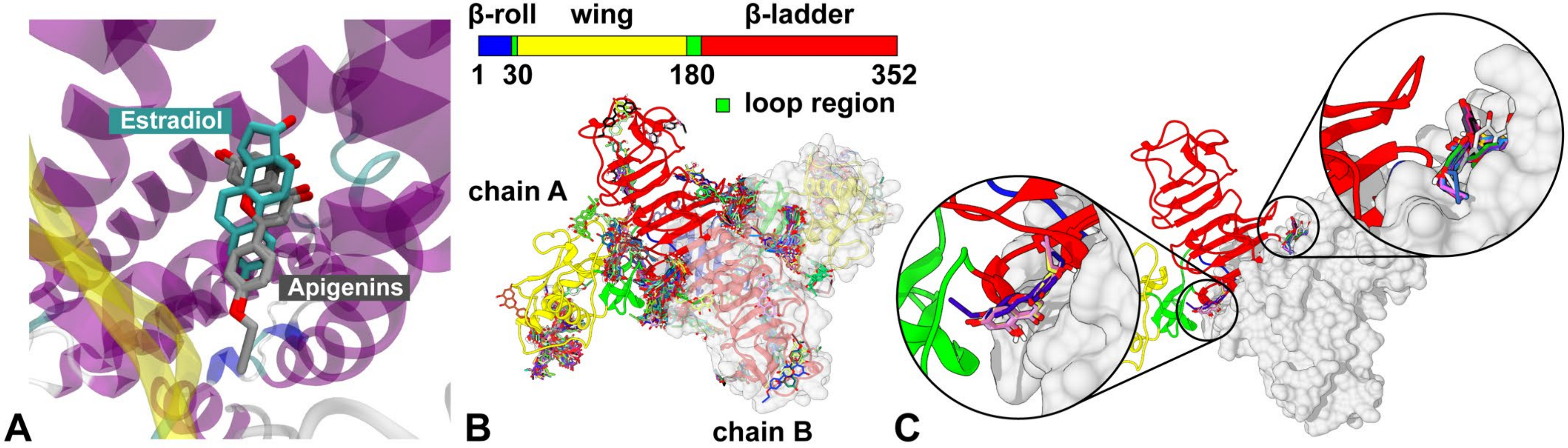


**Figure S1.** (**A**) structure overlay of Estradiol and apigenins (**B**,**C**) The 3D structure of dimeric NS1 of DENV2 with the top binding poses from 50 docking runs.

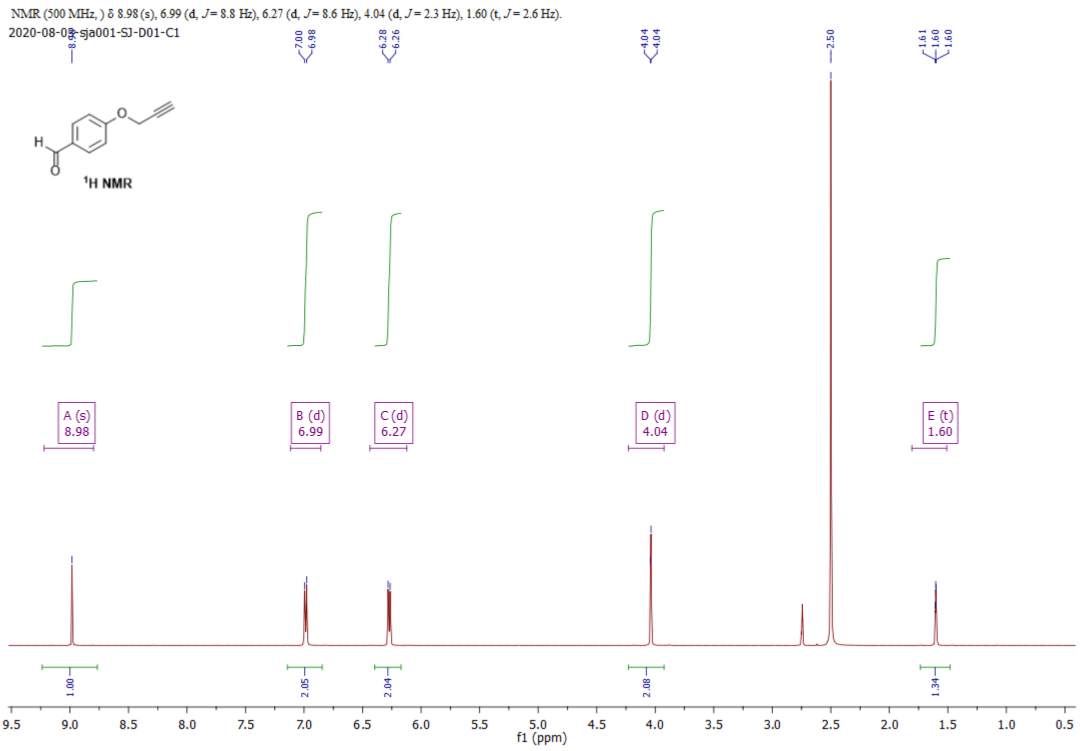


A

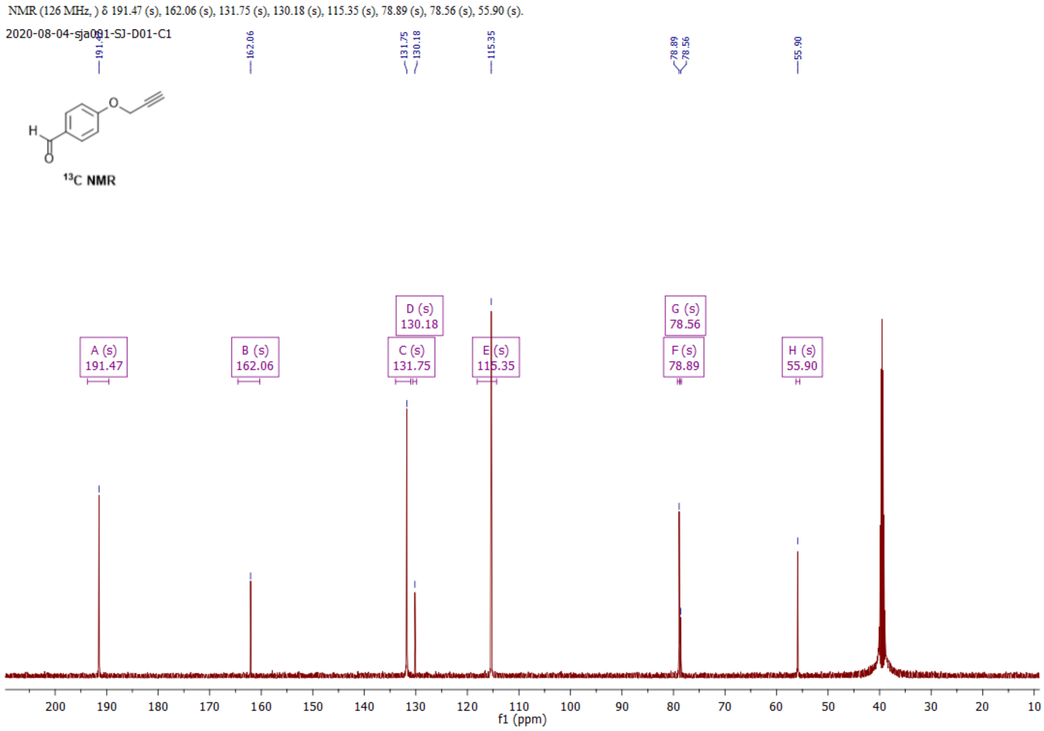


B

**Figure S2.** $^{1}$H (**A**) and $^{13}$C-NMR (**B**)of 4-(prop-2-yn-1-yloxy)benzaldehyde (**I**).

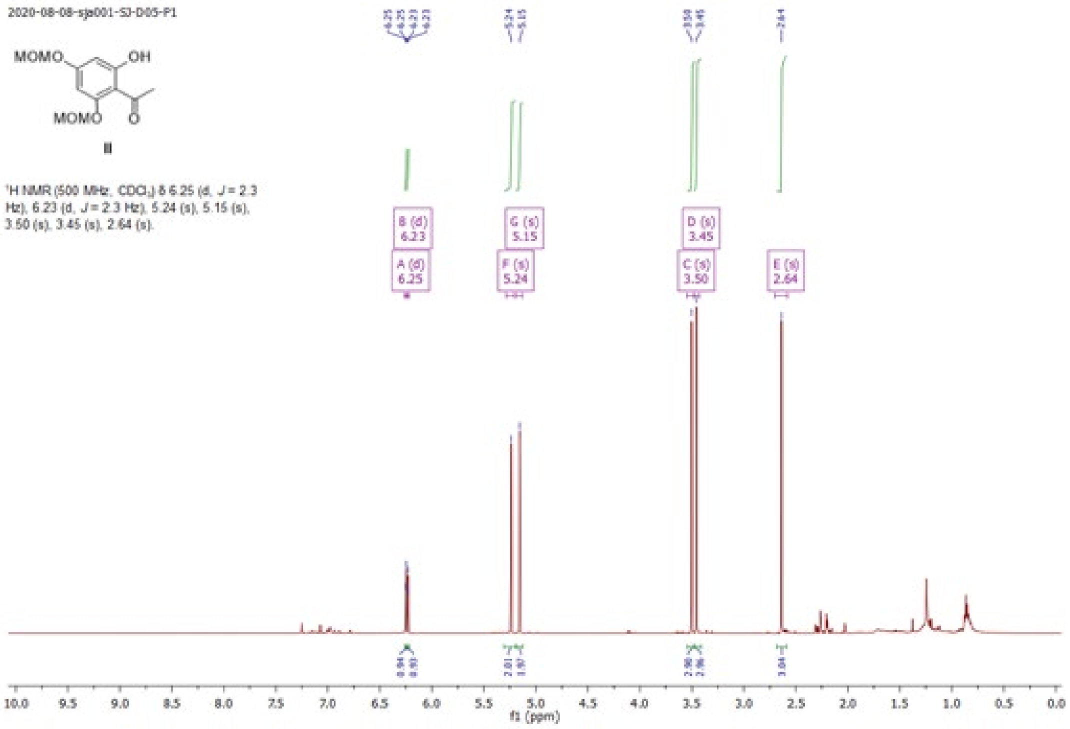


A

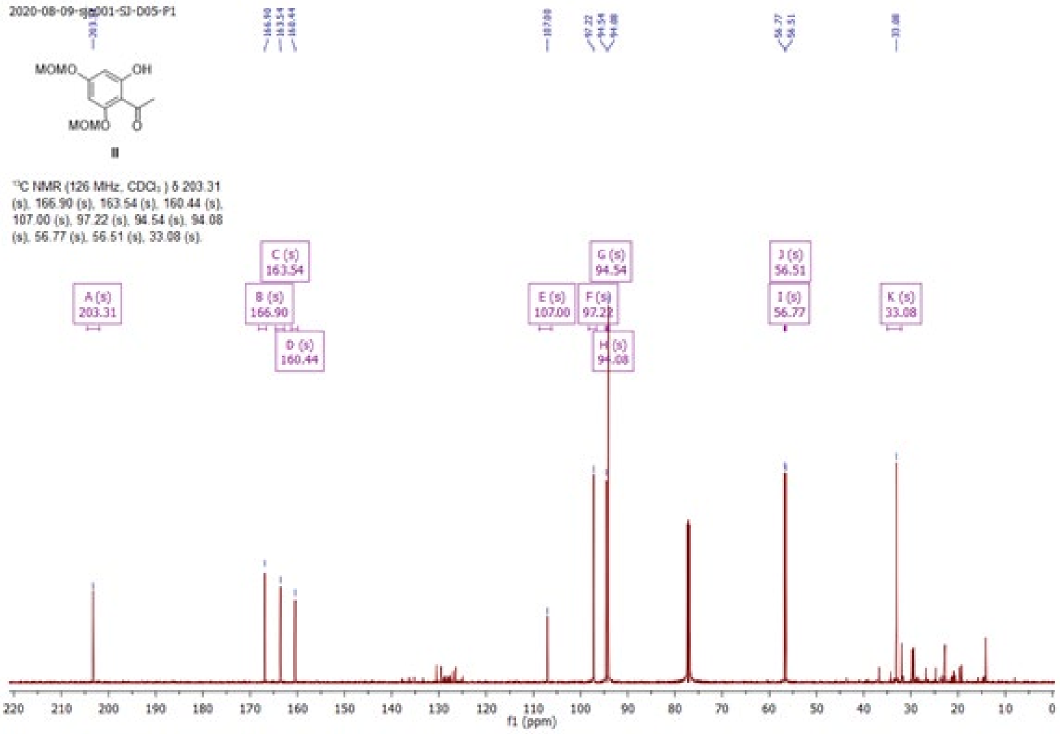


B

**Figure S3.** $^{1}$H (**A**) and $^{13}$C-NMR (**B**) of 1-(2-hydroxy-4,6-bis(methoxymethoxy)phenyl)ethan-1-one (**II**).

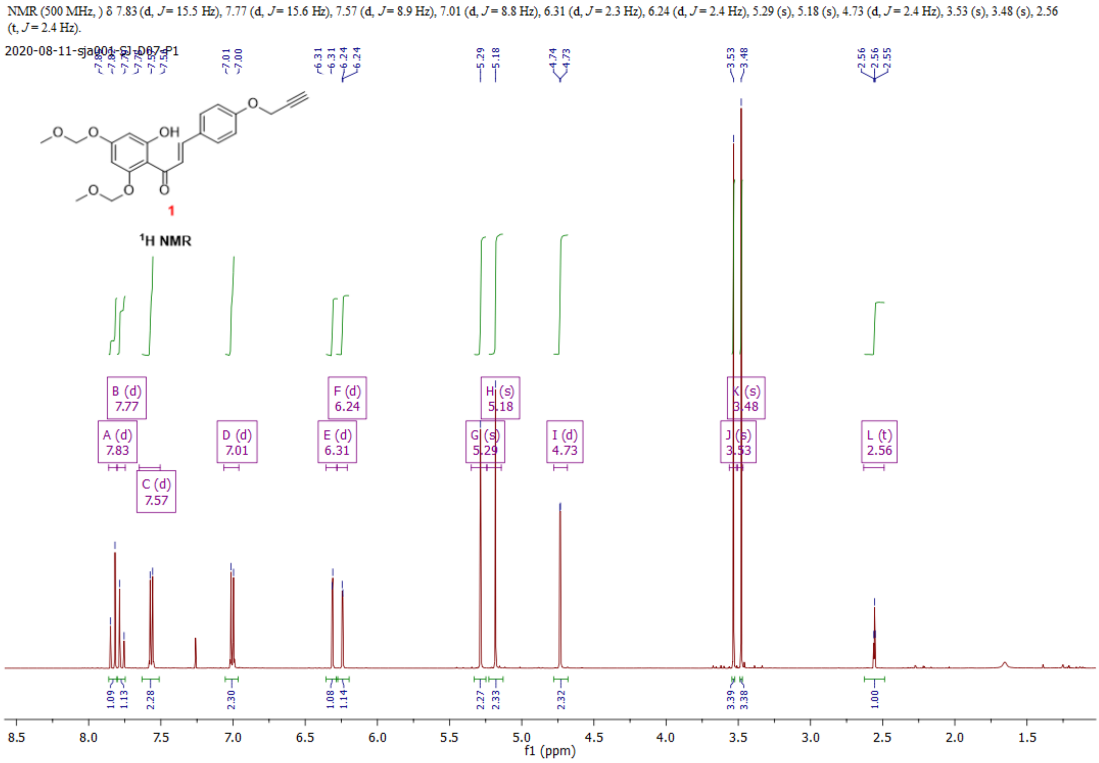


A

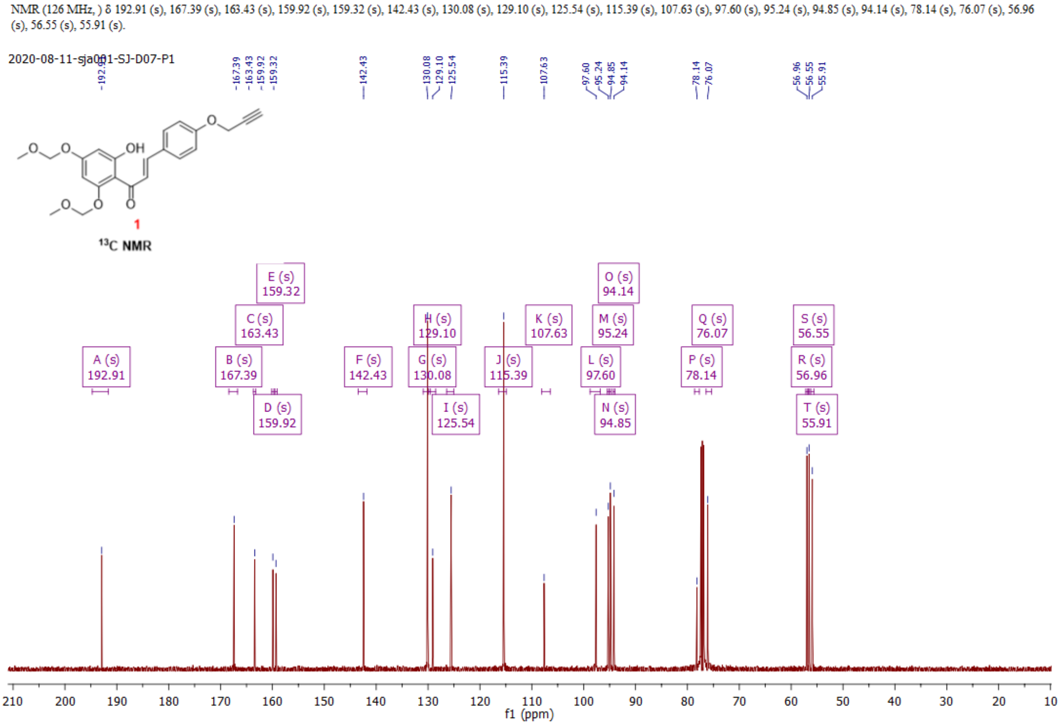


B

**Figure S4.** $^{1}$H (**A**) and $^{13}$C-NMR (**B**) of (*E*)-1-(2-hydroxy-4,6-bis(methoxymethoxy)phenyl)-3-(4-(prop-2-yn-1-yloxy)phenyl)prop-2-en-1-one (**1**).

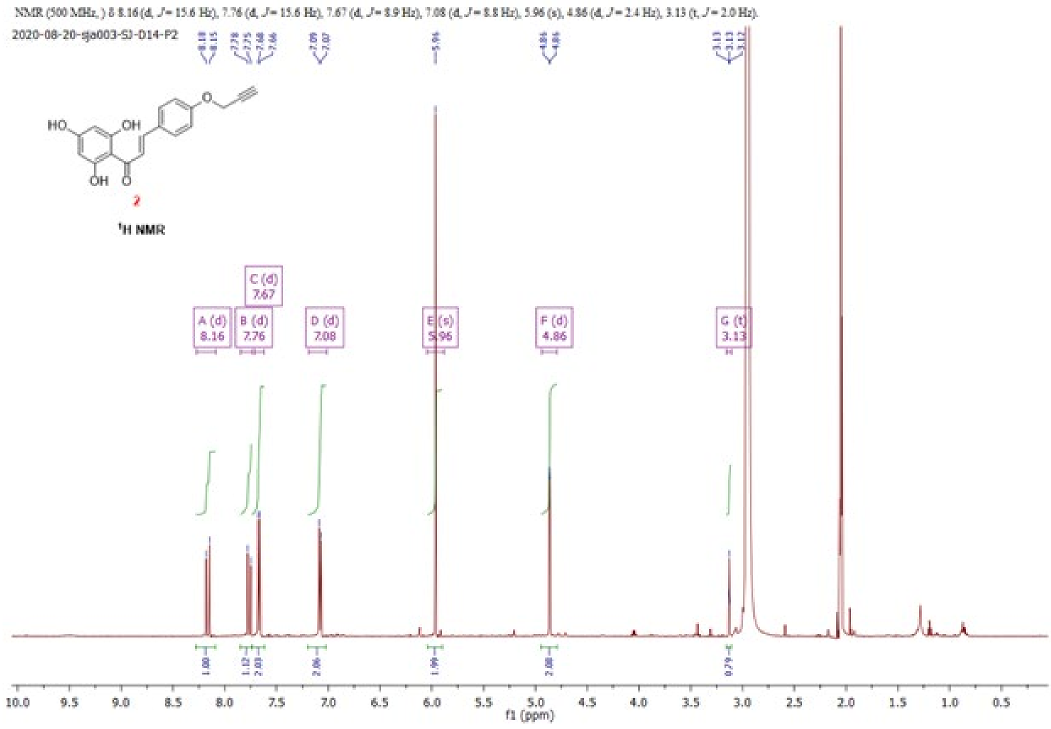


A

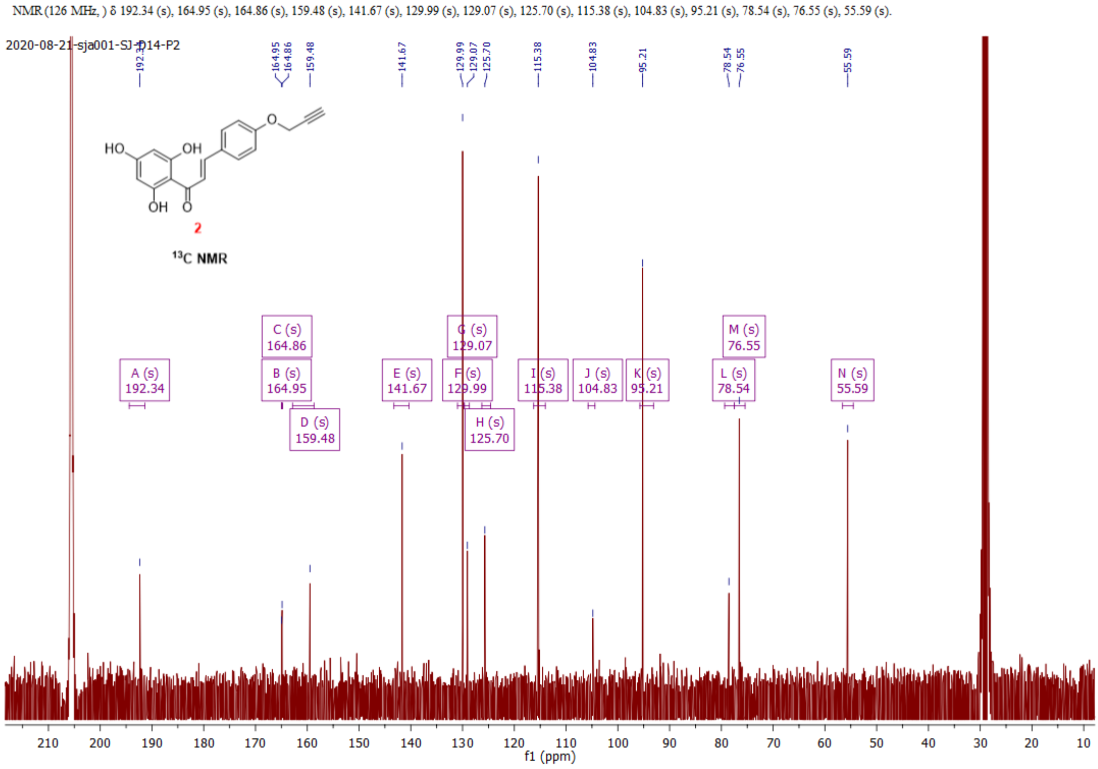


B

**Figure S5.** $^{1}H$ (**A**) and $^{13}C$-NMR (**B**) of 5,7-bis(methoxymethoxy)-2-(4-(prop-2-yn-1-yloxy)phenyl)-4*H*-chromen-4-one (**2**).

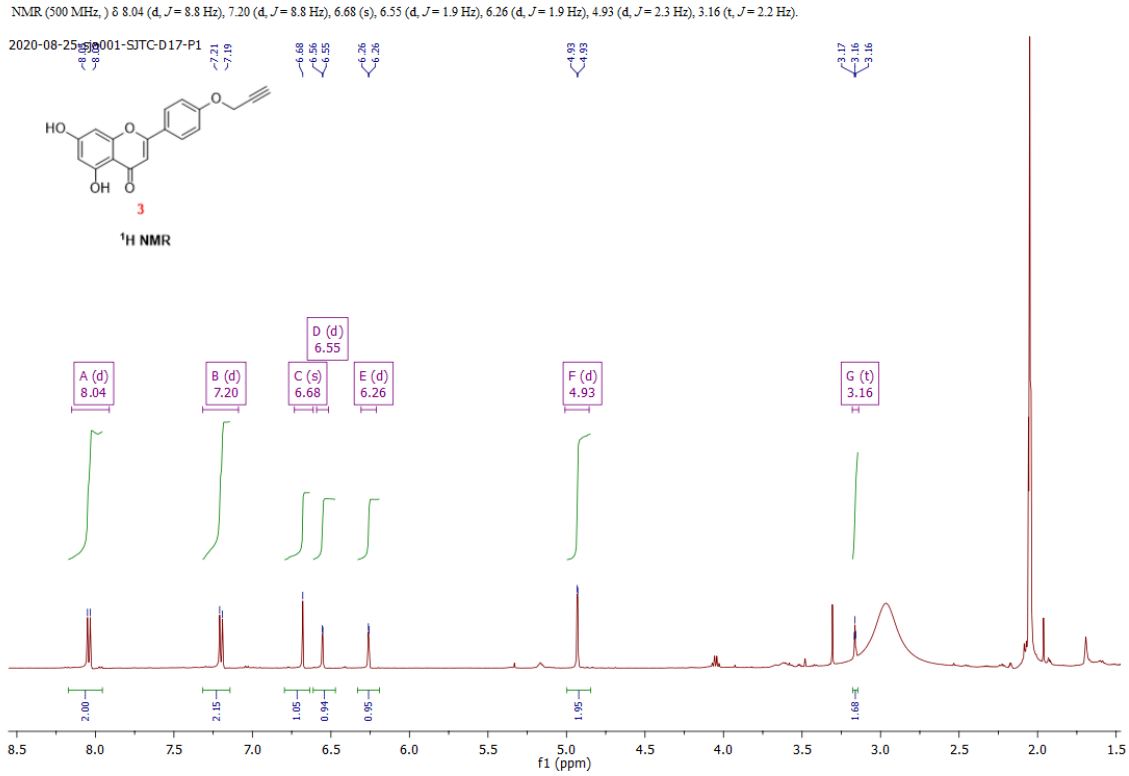


A

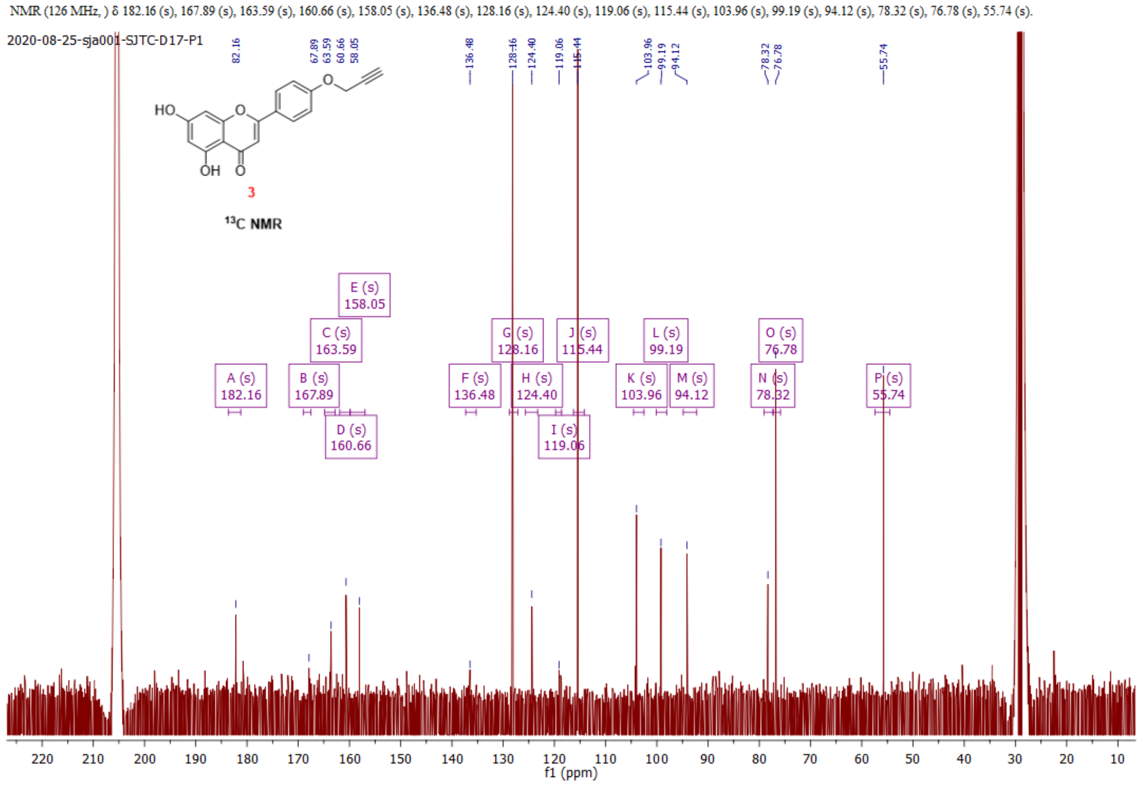


B

**Figure S6.** $^{1}$H (**A**) and $^{13}$C-NMR (**B**) of 5,7-dihydroxy-2-(4-(prop-2-yn-1-yloxy)phenyl)-4*H*-chromen-4-one (**3**).

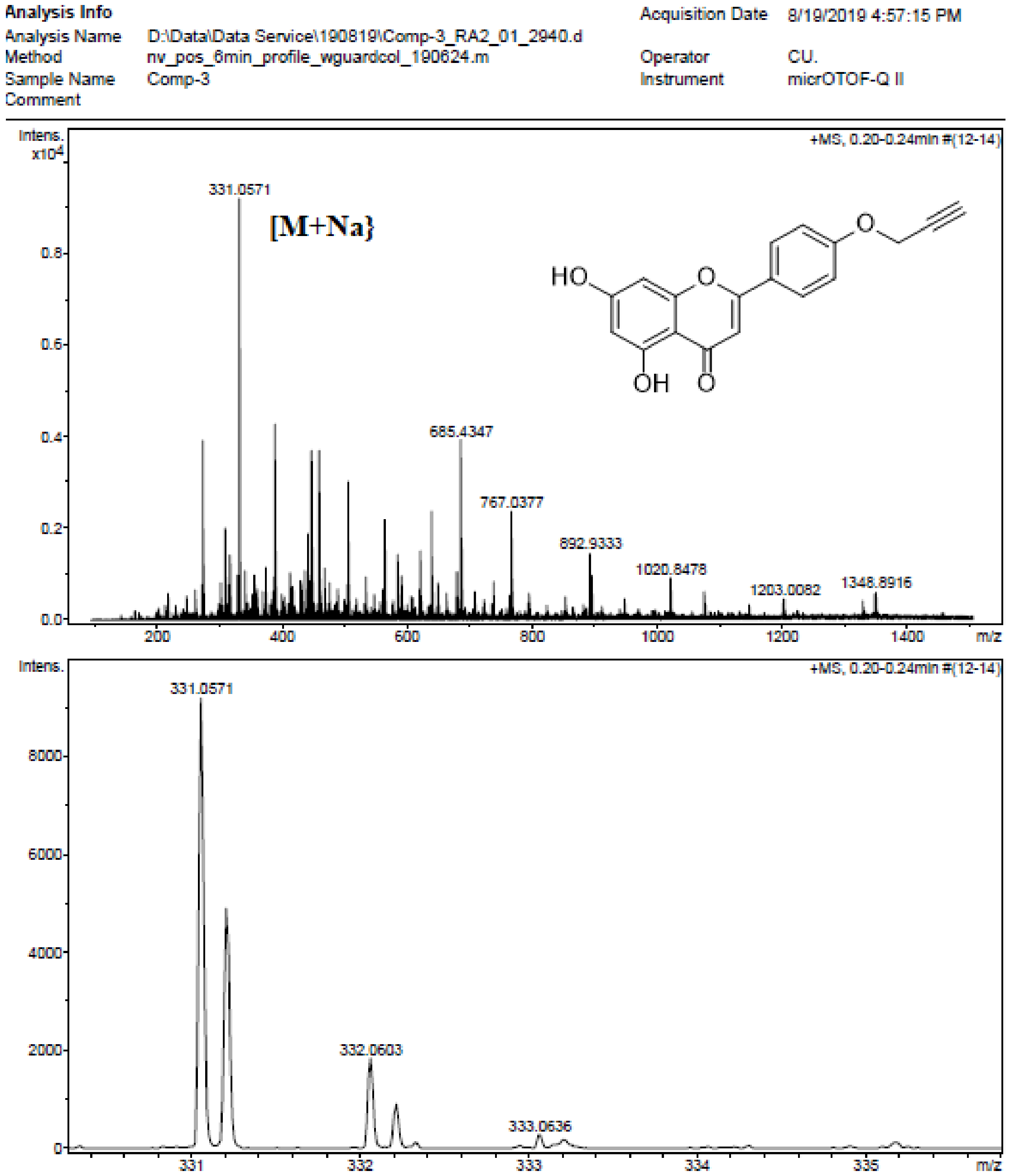


**Figure S7.** HRMS spectra of 5,7-dihydroxy-2-(4-(prop-2-yn-1-yloxy)phenyl)-4H-chromen-4-one (**3**).

## Alkyne-tagged apigenin

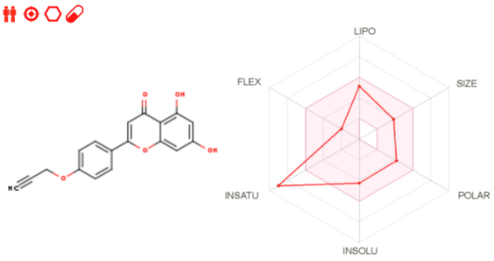


SMILES C#CCOc1ccc(cc1)c1cc(=O)c2c(o1)cc(cc2O)O

| Physicochemical Properties | |
|---|---|
| Formula | C18H12O5 |
| Molecular weight | 308.28 g/mol |
| Num. heavy atoms | 23 |
| Num. arom. heavy atoms | 16 |
| Fraction Csp3 | 0.06 |
| Num. rotatable bonds | 3 |
| Num. H-bond acceptors | 5 |
| Num. H-bond donors | 2 |
| Molar Refractivity | 86.23 |
| TPSA | 79.90 Å² |

| Lipophilicity | |
|---|---|
| Log $P_{o/w}$ (iLOGP) | 2.90 |
| Log $P_{o/w}$ (XLOGP3) | 3.46 |
| Log $P_{o/w}$ (WLOGP) | 2.96 |
| Log $P_{o/w}$ (MLOGP) | 1.18 |
| Log $P_{o/w}$ (SILICOS-IT) | 3.61 |
| Consensus Log $P_{o/w}$ | 2.82 |

| Water Solubility | |
|---|---|
| Log *S* (ESOL) | -4.25 |
| Solubility | 1.74e-02 mg/ml ; 5.65e-05 mol/l |
| Class | Moderately soluble |
| Log *S* (Ali) | -4.82 |
| Solubility | 4.67e-03 mg/ml ; 1.52e-05 mol/l |
| Class | Moderately soluble |
| Log *S* (SILICOS-IT) | -5.19 |
| Solubility | 1.98e-03 mg/ml ; 6.43e-06 mol/l |
| Class | Moderately soluble |

| Pharmacokinetics | |
|---|---|
| GI absorption | High |
| BBB permeant | No |
| P-gp substrate | No |
| CYP1A2 inhibitor | Yes |
| CYP2C19 inhibitor | No |
| CYP2C9 inhibitor | Yes |
| CYP2D6 inhibitor | Yes |
| CYP3A4 inhibitor | Yes |
| Log $K_p$ (skin permeation) | -5.72 cm/s |

| Druglikeness | |
|---|---|
| Lipinski | Yes; 0 violation |
| Ghose | Yes |
| Veber | Yes |
| Egan | Yes |
| Muegge | Yes |
| Bioavailability Score | 0.55 |

| Medicinal Chemistry | |
|---|---|
| PAINS | 0 alert |
| Brenk | 1 alert: triple_bond |
| Leadlikeness | Yes |
| Synthetic accessibility | 3.19 |

A

## Apigenin

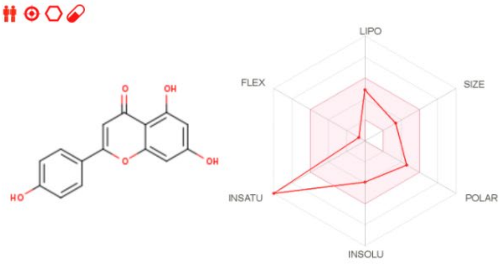


SMILES Oc1ccc(cc1)c1cc(=O)c2c(o1)cc(cc2O)O

| Physicochemical Properties | |
|---|---|
| Formula | C15H10O5 |
| Molecular weight | 270.24 g/mol |
| Num. heavy atoms | 20 |
| Num. arom. heavy atoms | 16 |
| Fraction Csp3 | 0.00 |
| Num. rotatable bonds | 1 |
| Num. H-bond acceptors | 5 |
| Num. H-bond donors | 3 |
| Molar Refractivity | 73.99 |
| TPSA | 90.90 Å² |

| Lipophilicity | |
|---|---|
| Log $P_{o/w}$ (iLOGP) | 1.89 |
| Log $P_{o/w}$ (XLOGP3) | 3.02 |
| Log $P_{o/w}$ (WLOGP) | 2.58 |
| Log $P_{o/w}$ (MLOGP) | 0.52 |
| Log $P_{o/w}$ (SILICOS-IT) | 2.52 |
| Consensus Log $P_{o/w}$ | 2.11 |

| Water Solubility | |
|---|---|
| Log *S* (ESOL) | -3.94 |
| Solubility | 3.07e-02 mg/ml ; 1.14e-04 mol/l |
| Class | Soluble |
| Log *S* (Ali) | -4.59 |
| Solubility | 6.88e-03 mg/ml ; 2.55e-05 mol/l |
| Class | Moderately soluble |
| Log *S* (SILICOS-IT) | -4.40 |
| Solubility | 1.07e-02 mg/ml ; 3.94e-05 mol/l |
| Class | Moderately soluble |

| Pharmacokinetics | |
|---|---|
| GI absorption | High |
| BBB permeant | No |
| P-gp substrate | No |
| CYP1A2 inhibitor | Yes |
| CYP2C19 inhibitor | No |
| CYP2C9 inhibitor | No |
| CYP2D6 inhibitor | Yes |
| CYP3A4 inhibitor | Yes |
| Log $K_p$ (skin permeation) | -5.80 cm/s |

| Druglikeness | |
|---|---|
| Lipinski | Yes; 0 violation |
| Ghose | Yes |
| Veber | Yes |
| Egan | Yes |
| Muegge | Yes |
| Bioavailability Score | 0.55 |

| Medicinal Chemistry | |
|---|---|
| PAINS | 0 alert |
| Brenk | 0 alert |
| Leadlikeness | Yes |
| Synthetic accessibility | 2.96 |

B

**Figure S8.** Pharmacokinetics, drug-likeness and medicinal chemistry by SwissADME for alkyne-tagged apigenine (**A**) and Apigenin (**B**).
